# Metabolomics Approach to Identify Biomarkers of Acute and Subacute Mastitis in Milk Samples: A Pilot Case–Control Study

**DOI:** 10.3390/metabo14100566

**Published:** 2024-10-21

**Authors:** Paola Quifer-Rada, Laia Aguilar-Camprubí, Sara Samino, Nuria Amigó, Oria Soler, Alba Padró-Arocas

**Affiliations:** 1 LactApp Women Health, 08014 Barcelona, Spain; paola@lactapp.es (P.Q.-R.); laia@lactapp.es (L.A.-C.); 2 Department of Endocrinology & Nutrition, Biomedical Research Institute Sant Pau, Hospital de la Santa Creu i Sant Pau, 08025 Barcelona, Spain; 3 CIBER of Diabetes and Associated Metabolic Diseases, 28029 Madrid, Spain; ssamino@biosferteslab.com (S.S.); namigo@biosferteslab.com (N.A.); 4 Biosfer Teslab, 43206 Reus, Spain; oria.soler@urv.cat; 5 Department of Basic Medical Sciences, University Rovira I Virgili, IISPV, 43204 Reus, Spain

**Keywords:** breast feeding, human milk, mastitis, biomarkers, lipidomics

## Abstract

**Background and aims**: Mastitis is one of the main complications during breastfeeding and contributes to the cessation of breastfeeding. However, the etiopathogenesis and diagnosis of mastitis are complex and not yet well defined. We aimed to identify metabolic and lipidic changes in human milk during acute and subacute mastitis in order to detect potential biomarkers of mastitis. **Methods:** We conducted a pilot case–control study including 14 breastfeeding women with acute mastitis, 32 with subacute mastitis symptoms, and 19 without any mastitis symptoms (control). Milk samples were collected and analyzed by proton nuclear magnetic resonance (H-NMR) for metabolomics analysis. To assess the association between the significant metabolites and lipids and the development of acute and subacute mastitis, multi-adjusted logistic regression models were developed. **Results:** The NMR-based metabolomics approach was able to identify and quantify a total of 40 metabolites in breast milk samples. After adjusting for confounding variables, acute mastitis was significantly associated with acetate (OR 3.9 IC 1.4–10.8), total cholesterol (OR 14 CI 3.2–62), esterified cholesterol (OR 3.3 CI 1.9–5.8), and sphingomyelin (OR 2.6 CI 1.2–5.8). The other metabolites presented weak association (OR < 2.5). Subacute mastitis was significantly associated with glutamine, lysophosphatidylcholine, phosphatidylcholine, plasmalogen, and total polyunsaturated fatty acids, but only cholesterol showed a strong association (OR > 2.5) with an OR of 2.6 (IC 1.1–6.6). **Conclusions**: Metabolic alteration in breast milk occurs during a process of both acute and subacute mastitis. Acetate, esterified cholesterol, lysophostidylcholine, and polyunsaturated fatty acids increased in both acute and subacute mastitis. However, according to the multi-adjusted regression logistic models, the candidate biomarkers for acute and subacute mastitis are cholesterol, lysophosphatidylcoholine, phosphatidylcholine, plasmalogen, and polyunsaturated fatty acids.

## 1. Introduction

It has been demonstrated that breastfeeding is the optimal feeding for infants. Major professional health organizations, such as the WHO or the American Association of Pediatrics (AAP), currently promote breastfeeding by recommending exclusive breastfeeding until the baby is 6 months old and supporting continued breastfeeding, along with complementary foods introduced at 6 months, as long as mutually desired by mother and child for 2 years or beyond [1,2].

Breastfeeding has a positive impact on both the mother’s and the infant’s health and is directly associated with decreases in diseases such as cancer, diabetes, cardiovascular disease, and inflammatory and infectious diseases [3,4,5].

However, according to UNICEF, at 6 months, only two out of five infants continue to receive exclusive breastfeeding [6]. The main factors that influence the abandonment of breastfeeding are returning to work, the feeling of insufficient milk supply, problems in breastfeeding, and breast complications like mastitis, with pain being its main cause [7].

Recent studies have linked breastfeeding pain to inflammation in the mammary gland (mastitis) caused by a process of dysbiosis [8,9]. The incidence of mastitis ranges from 3 to 33% and appears most frequently in the first 3 months of lactation [10]. The etiopathogenesis of mastitis is complex and not yet well defined, but it has been observed that the most important factors for the development of mastitis are milk stasis (milk retention) and bacterial overgrowth [9].

Three different types of mastitis have been described: acute mastitis (AM), subacute mastitis (SAM), and subclinical mastitis. Nevertheless, the clinical definitions of these complications are still controversial. AM is characterized by an inflammation of mammary lobules caused by the overgrowth of mostly *Staphylococcus aureus* and causes acute symptoms in the breast such as pain, tenderness, swelling, and hot and reddened breasts along with systemic symptoms such as malaise, arthromyalgia, fever, chills, and headaches [9].

SAM causes deep pain in the breast during or after feeding but does not cause any visible breast or systemic symptoms [9]. In clinical practice, SAM is often misdiagnosed as noninfectious inflammation or breast candidiasis [11,12], causing chronic pain in lactating women that may lead to early weaning.

In order to increase breastfeeding rates, it is necessary to find factors related to the progression of mastitis in breastfeeding women. The identification of metabolic biomarkers could revolutionize the early diagnosis and management of mastitis, offering a practical approach to mitigating breastfeeding pain and promoting long-term breastfeeding success.

Metabolomics is an emerging technology that is revolutionizing the field of medicine since it enables the measurement of all metabolites and low-molecular-weight molecules that are integrated within a biological matrix, so the technique provides a complete picture of the biological and pathological processes and metabolic pathways that are being altered. The biomarker panels obtained using this analytical platform can be used for accurate disease diagnosis and prognosis. While this approach has been successfully used in breast milk samples in clinical studies with different research goals [13,14,15], the use of this technology to specifically investigate mastitis-related metabolic alterations is sparse. To date, no studies have thoroughly explored the metabolic and lipidic changes in human breast milk during both acute and subacute mastitis.

In this context, the present study aims to perform a pilot study to identify potential metabolic biomarkers for both acute and subacute mastitis that may serve as early indicators of mastitis, facilitating timely intervention and potentially reducing breastfeeding discontinuation due to pain.

## 2. Experimental Section

### 2.1. Study Design and Participants

We performed a case–control pilot study to identify predictive biomarkers of progression to acute and subacute mastitis in lactating women.

Mastitis was diagnosed clinically using the definitions of the PRIOAM guidelines [16] that are in accordance with the latest protocols on mastitis of The Academy of Breastfeeding [9].

AM was defined as acute symptoms in the breast such as tenderness or pain, swelling, heat, redness, and blocked breast ducts or milk retention along with general symptoms of myalgia, headache, nausea, or chills [9,16]. Moreover, participants in this group needed to have fever (>38 °C) to confirm the infection.

SAM was defined as deep breast pain or a burning or needle-like pain in the nipple during or after feeding that did not resolve after evaluation and intervention by a professional lactation consultant. Milk blebs may be also present, and the patient does not present any general symptoms or fever. The physical examination of the breast is usually normal [9,16].

**Figure 1:**
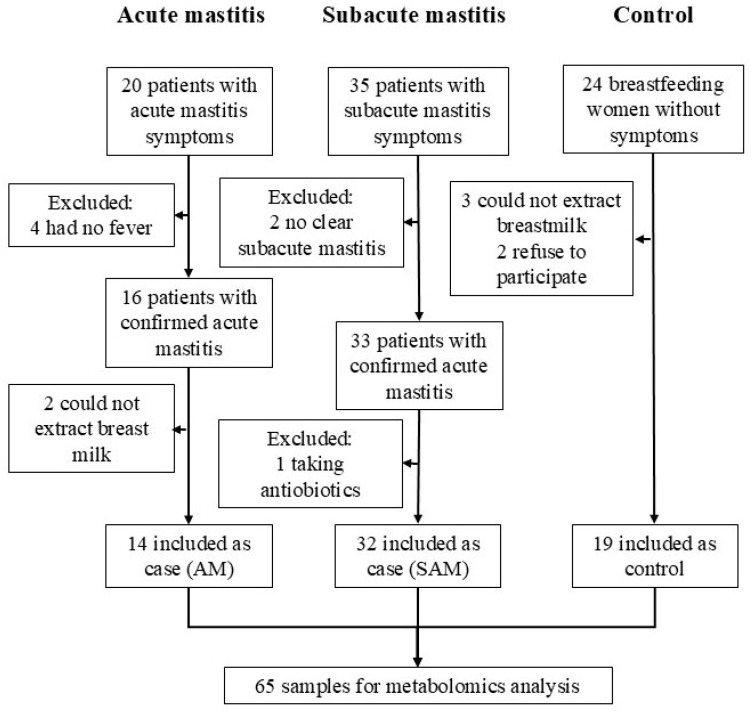
Flow chart of the study and the participants’ recruitment.

In total, 14 breastfeeding women with AM, 32 with SAM symptoms, and 19 without any mastitis symptoms (control) were recruited through a social media call using *LactApp* channels. *LactApp* is a mobile health app aiming to support breastfeeding [17,18] and has a large community of lactating mothers (Figure 1).

The inclusion criteria were breastfeeding women with healthy full-term infants older than 4 weeks and younger than 6 months and who have freely given their consent to participate in the study.

The exclusion criteria were immunosuppressed women such as HIV patients and women undergoing treatment with immunosuppressants or chemotherapy; women who initiated antibiotic treatment; women who exclusively pumped milk; mothers with babies suffering from any syndrome, cleft lip, or facial malformations; mothers of twins or triplets or following tandem breastfeeding; and women with premature birth.

### 2.2. Sample Collection

Participants with and without mastitis symptoms willing to participate were asked to contact the investigators through an online form. Participants who met the inclusion and exclusion criteria were scheduled for a video call with a midwife to perform a clinical examination to confirm the presence of acute and subacute mastitis or the absence of symptoms for control samples.

Women included in the study received a kit of collection material (sterile pots and pre-labeled tubes) to collect their own milk at home and were asked to immediately freeze the milk samples. Women also received a guide of how to manually express milk correctly and, if needed, they were also helped though a video call. Frozen milk samples were picked up from the participant’s home by a courier company specialized in cold shipments. Samples were stored in *LactApp* facilities until being sent to Biosfer Lab, where they were stored at −80 °C prior to metabolomics analysis.

Clinical data of the participants were collected during the online appointment with the midwife, and sociodemographic, lactation experience, pregnancy, and diet data were collected using an online survey.

### 2.3. Ethics

The research protocol was approved by the ethics committee of Blanquerna Ramon Llull University (CER-FCSB num. 2021-01-01) in April 2021.

All participants received the participant information sheet and gave their written consent.

### 2.4. H-NMR Metabolomics Analysis

In this study, we selected H-NMR as the primary metabolomics technology to analyze breast milk samples instead of liquid chromatography–mass spectrometry (LC-MS). While LC-MS is highly sensitive and capable of detecting low-abundance metabolites, H-NMR is more robust and reproducible and does not require extensive sample preparation. Moreover, H-NMR allows direct and reliable quantification of metabolites in a single analysis, providing comprehensive results more efficiently. Most importantly, our goal was to identify biomarkers present at relatively higher concentrations in breast milk, which can later be measured using routine, accessible technologies in clinical laboratories. By focusing on robust and quantifiable biomarkers, we aim to facilitate the development of simpler diagnostic tools for mastitis, suitable for routine clinical use.

Frozen milk samples were shipped on dry ice to Biosfer Teslab (Reus, Spain) for the metabolomics analysis. Milk samples were extracted using the Folch [19] method with slight modifications. Briefly, 600 µL of milk was extracted by adding 3 mL of a cold mixture of chloroform/methanol (2:1 *v*/*v*). The resulting suspension was vortexed and subsequently added 400 µL of ultrapure water. Samples were vortexed again and 1 mL of chloroform was added. The resulting mixture were centrifuged (4000 rpm, 10 min at 4 °C) and 1.8 mL of aqueous and organic phase was collected separately for drying, until evaporation, in a SpeedVac (Thermo Fisher, Rockford, IL, USA).

The aqueous phase was resuspended in 650 µL of deuterated water (D_2_O) containing 2.32 mM of Trimethylsilylpropionic acid sodium salt (TSP) and transferred to a 5mm H-NMR tube. The organic phase was resuspended in 700 µL of deuterated chloroform/methanol/water (CDCl_3_:CD_3_OD:D_2_O—16:7:1) and transferred to a 5mm H-NMR tube.

^1^H-NMR spectra were recorded on a Bruker Avance III 600 spectrometer coupling a Sample Jet operating at a proton frequency of 600 MHz. The acquisition temperature of the samples was 300 K for the aqueous phase and 286 K for the organic phase. For the aqueous phase, one-dimensional ^1^H pulse experiments were carried out using the nuclear Overhauser effect spectroscopy (NOESY)–presaturation sequence to suppress the residual water peak at around 4.7 ppm. For the organic phase, a 90° pulse with water presaturation sequence (zgpr) was used. For the aqueous metabolites, a NOESY pulse sequence was applied at a temperature of 300 K. A total of 64 scans were performed for all samples, and the receiver gain was optimized and set at 181. The relaxation delay used for the aqueous spectra was 5 s, which was specifically optimized for the detection of aqueous metabolites.

For the lipid metabolites, the zgpr pulse sequence was employed at a temperature of 286 K, with 64 scans performed for all samples, and the gain set at 144. The relaxation delay for the lipid spectra was also 5 s.

Both relaxation times were carefully optimized to ensure accurate metabolite detection in their respective phases.

**Figure 2:**
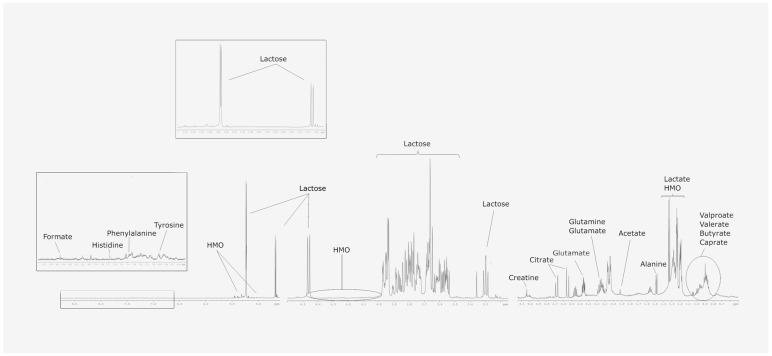
One-dimensional proton spectrum of the aqueous phase of the breast milk samples.

The acquired spectra were phased, baseline-corrected and referenced before performing the automatic metabolite profiling. For the aqueous phase, metabolomic profiling was performed by using an adaptation of Dolphin [20] (Figure 2). For the organic phase, lipid families were profiled by using an adaptation of LipSpin [21]. Resonance assignments were performed using Chenomx, HMDB [22], and on the basis of the literature [23].

### 2.5. Statistical Analysis

Power analysis was conducted using the MetSizeR application (R version 4.3.3), which is specifically designed for metabolomics studies. Given the high-dimensional nature of metabolomics data, we simulated power based on several key parameters. These included 50 spectral bins, a proportion of 0.5 significant bins, and a model based on Probabilistic Principal Component Analysis (PPCA). We aimed to control for a false discovery rate (FDR) of 0.05 and set a minimum sample size of 4 per group. The simulation results indicated that 36 samples would be required to detect significant metabolic changes under these conditions (12 sample per group).

Baseline characteristics of the participants among the three groups of the study were compared. Categorical variables were compared using a Chi-square test. Continuous variables were tested for normality using a Shapiro–Wilk test, and since the distribution of the variables was not normal, a Kruskal–Wallis test for multiple groups was used.

To assess lipidomic and metabolomic changes between control and AM and control and SAM samples, an unpaired U Mann–Whitney–Wilcoxon nonparametric test was used due to the low number of participants (less than 30 in each group).

Furthermore, to assess the association between the significant metabolites and lipids and the development of acute and subacute mastitis, logistic regression was performed. Logistic regression models were performed unadjusted and further adjusted based on the following confounding variables: infant ankyloglossia (presence or absence), autoimmune disease, anemia, gestational diabetes, thyroid disease, smoking habit, antibiotics taken during pregnancy, antibiotics administered during birth, antibiotics taken during breastfeeding, antifungals taken during breastfeeding, and probiotics taken during breastfeeding. *p*-values were further adjusted by FDR.

Partial Least Squares Discriminant Analysis (PLS-DA) was conducted using the mixOmics package in R to assess the differentiation between the AM and control groups, as well as the SAM and control groups, based on the metabolites identified through logistic regression. To evaluate the model’s performance, we implemented cross-validation using M-fold validation with 5 folds and 100 repetitions to ensure robustness and reproducibility. The classification error rates were subsequently extracted for analysis.

## 3. Results

### 3.1. Participant Characteristics

The mean age of the women who participated in the study was 34 years (minimum age 27 years, maximum age 43 years). Most of the participants exclusively breastfed (90.7%) and this was their first breastfeeding experience (75.3%). All participants had a full-term pregnancy with a mean birth gestational week of 39 weeks (minimum 37 gestational weeks, maximum 42 gestational week). The infants’ mean age was 3.1 months (minimum infant age 1 month, maximum infant age 6 months).

There was no significance difference in sociodemographic and clinical data related to mastitis development according to the study groups (Table 1) except for probiotic intake during pregnancy, which was significantly more frequent in the SAM group (96.9%), followed by the CT (84.2%) and AM (71.4%) groups (*p*-value = 0.046), and mode of birth, for which c-section was significantly more frequent in the CT group (36.8%) followed by the AM group (14.3%) and SAM group (6.3%) (*p*-value = 0.018).

### 3.2. Metabolites Identified in Human Milk Samples

**Figure 3:**
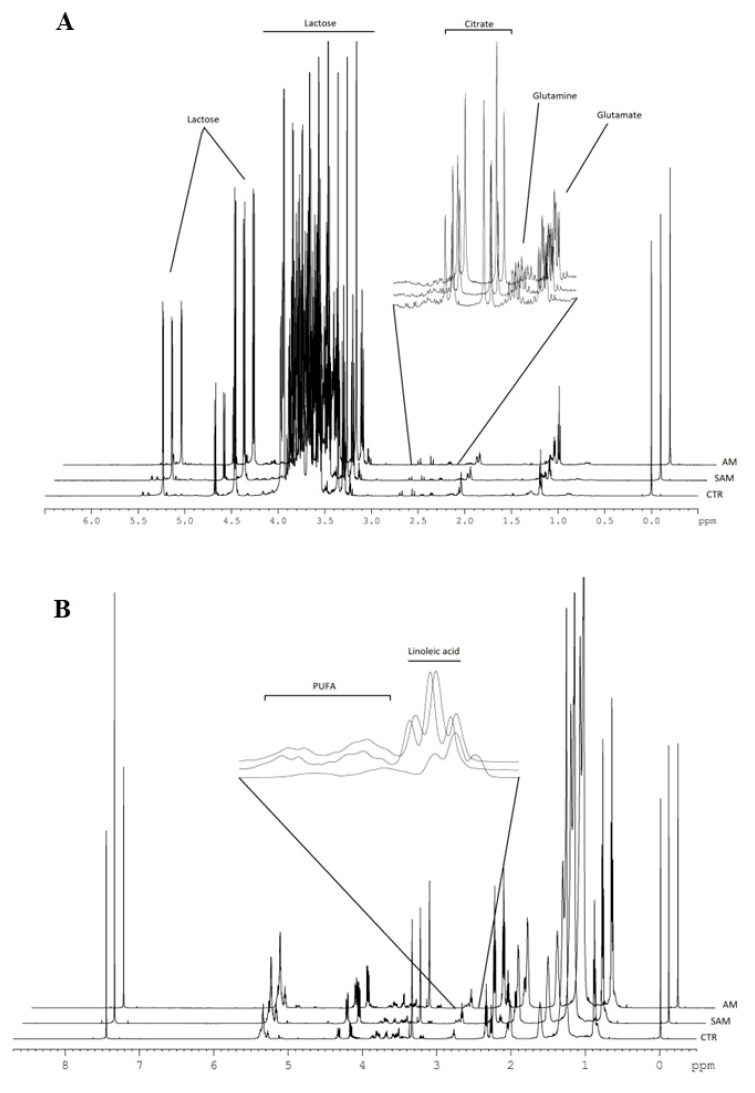
Stacked plot of 1H NMR spectra of the aqueous (**A**) and lipidic (**B**) phases of breast milk samples.

Figure 2 shows the identification of the metabolites in the aqueous phase in a one-dimensional proton spectrum and Figure 3 shows the stacked plot of 1H NMR spectra of the aqueous and lipidic phases.

The NMR-based metabolomics approach was able to identify and quantify a total of 40 metabolites in breast milk samples (Table 2), including carboxylic acids, dicarboxylic acids, amino acids, amines, gamma-keto-acids, cholines, disaccharide, long-chain fatty acids, very-long-chain fatty acids, phosphocholines, phosphatidylcholine, phosphatidylethanolamine, plasmalogen, polyunsaturated fatty acids, sphingomyelin, triglycerides, and cholesterol.

### 3.3. Changes in Metabolite Concentrations in Human Milk during Mastitis

The Wilcoxon test revealed significant changes in the concentration of certain metabolites in AM and SAM milk samples (Table 2).

Compared to healthy milk samples, the AM process significantly increased the levels of acetate (mean increase 1.54 µmol/L, *p*-value = 0.001), citrate (mean increase 28.3 µmol/L, *p*-value = 0.027), creatine (mean increase 1.64 µmol/L, *p*-value = 0.049), total cholesterol (mean increase 1.84 µmol/L, *p*-value < 0.001), esterified cholesterol (mean increase 0.95 µmol/L, *p*-value < 0.001), isoleucine (mean increase 1.28 µmol/L, *p*-value = 0.036), lactate (mean increase 53.1 µmol/L, *p*-value = 0.001), lysophosphatidylcholine (mean increase 0.23 µmol/L, *p*-value < 0.001), phosphatidylcholine (mean increase 0.52 µmol/L, *p*-value < 0.001), phosphatidylethanolamine (mean increase 2.24 µmol/L, *p*-value = 0.030), plasmalogen (mean increase 49.5 µmol/L, *p*-value < 0.001), total polyunsaturated fatty acids (mean increase 0.5 µmol/L, *p*-value < 0.001), and total triglycerides (mean increase 468.4 µmol/L, *p*-value = 0.001) and significantly decreased the total sphingomyelin (mean decrease −6.2 µmol/L, *p*-value = 0.006) and threonine (mean decrease −48.7 µmol/L, *p*-value = 0.011).

SAM also altered the expression of some metabolites, but at a much lower level. Compared to control samples, SAM significantly increased acetate (mean increase 0.34 µmol/L, *p*-value = 0.011), esterified cholesterol (mean increase 0.28 µmol/L, *p*-value = 0.012), lysophosphatidylcholine (mean increase 0.02 µmol/L, *p*-value = 0.028), O-Phosphocholine (mean increase 12.5 µmol/L, *p*-value = 0.003), and total polyunsaturated fatty acids (mean increase 0.08 µmol/L, *p*-value = 0.002) and significantly decreased triglycerides (mean decreased −470.3 µmol/L, *p*-value = 0.001) and glutamine (mean decrease −18.53 µmol/L, *p*-value = 0.004).

Logistic regressions were then performed to evaluate the strength of the association between the significant metabolites and the development of acute and subacute mastitis (Table 3). After adjusting for confounding variables, AM was significantly associated with acetate, total cholesterol, esterified cholesterol, glutamine, lysophosphatidylcholine, phosphatidylcholine, plasmalogen, total polyunsaturated fatty acids, total triglycerides, and total sphingomyelin.

However, the strongest association was found between acetate (OR 3.9 IC 1.4–10.8) total cholesterol (OR 14 CI 3.2–62), esterified cholesterol (OR 3.3 CI 1.9–5.8) and sphingomyelin (OR 2.6 CI 1.2–5.8). The other metabolites presented weak association (OR < 2.5).

SAM was significantly associated with lysophosphatidylcholine, phosphatidylcholine, plasmalogen, and total polyunsaturated fatty acids, but only total cholesterol showed a strong association (OR > 2.5) with an OR of 2.6 (IC 1.1–6.6).

**Figure 4:**
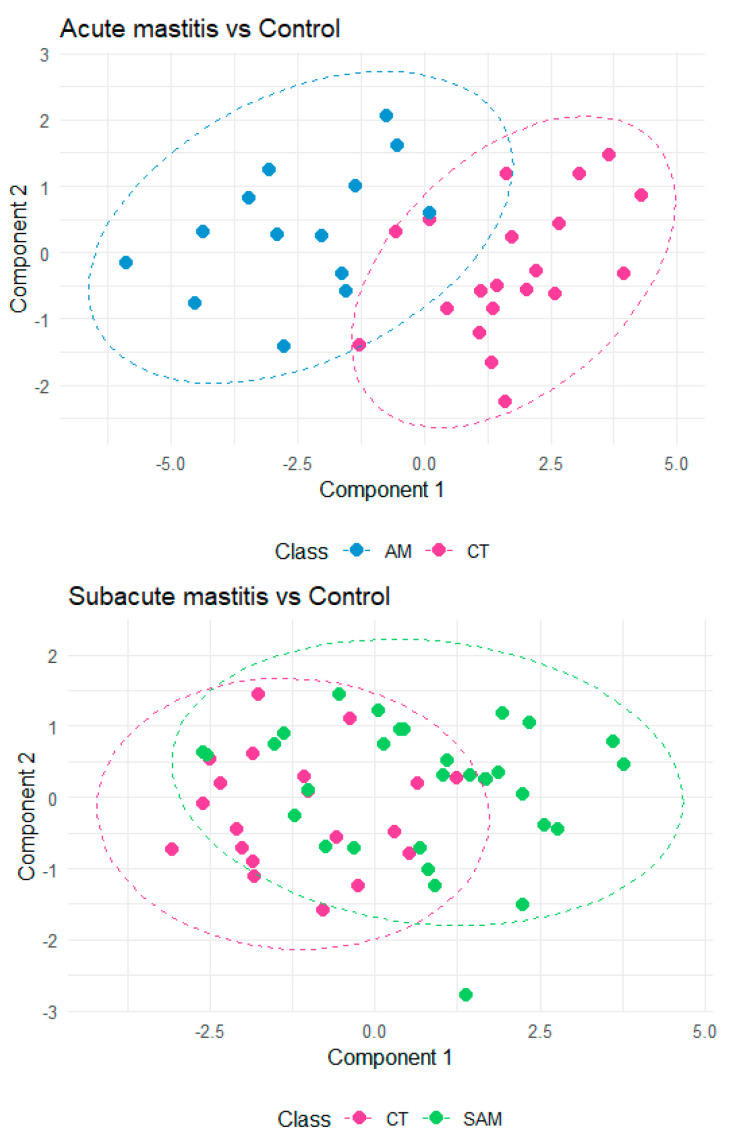
PLS-DA on the metabolites significantly associated with AM and SAM as identified by logistic regression.

PLS-DA was conducted on the metabolites significantly associated with AM and SAM as identified by logistic regression, comparing AM and control samples as well as SAM and control samples (Figure 4). The PLS-DA plot for the AM vs. control comparison demonstrates clear separation and effective classification between the two groups, which is supported by the selected metabolites. The centroid classification error rate for the AM vs. control PLS-DA model was 0.11, indicating high accuracy and predictive classification. In contrast, the SAM vs. control PLS-DA model exhibited lower accuracy, with a centroid classification error rate of 0.28, suggesting that nearly 30% of the samples were not correctly classified into their respective groups.

## 4. Discussion

The main aim of our study was to identify metabolic changes in human milk specific to mastitis in order to detect specific biomarkers of mastitis, both AM and SAM.

It is well known that human milk composition changes during an acute or subacute mastitis process due to the inflammatory state of the gland. For example, it has been reported that the lactose content decreases [24,25,26] and the content of minerals, such as sodium and chlorine, increases due to an increased permeability of the blood–milk barrier [24,25,26,27]. Furthermore, it has been reported that some proteins and inflammatory cytokines are over-expressed in both human and animal milk, such as tumor necrosis factor alpha (TNFα), interleukins IL1, IL2, IL6, IL8 and IL12, and immunological factors like lactoferrin, IFN-ɣ, IP-10, haptoglobin, amyloid A and C-reactive protein [25,28,29,30,31,32,33,34,35].

Our results also show some metabolic changes that may indicate shifts in some metabolic pathways in the breast.

According to the results of the Wilcoxon test, acetate is significantly increased in mastitic breast milk, in both AM and SAM, whereas lactate is only significantly increased during AM. Acetate and lactate are end products of the catabolism of glucose by bacteria [36,37,38,39] such as *S. aureus* and *S. epidermidis*, the main bacterial species that cause AM and SAM, respectively. AM and SAM are not produced by the same bacterial species; thus, the end-point products of the glucose metabolism can be different and may explain why AM milk has significantly higher levels of lactate compared to healthy milk but SAM milk does not. *S. aureus* is the main species that causes AM and uses pyruvate according to the growth conditions; under anaerobic growth, pyruvate is reduced to lactic acid, whereas during aerobic growth, pyruvate undergoes oxidative decarboxylation which leads to the generation of ATP and acetate [39]. Our results are in agreement with other studies conducted in cows that also showed an increase in end products of the bacterial catabolism of glucose during an episode of mastitis, such as lactic acid [40,41], acetate [42], and butyrate [42].

Metabolites related to amino acid biosynthesis and energy metabolism were also increased during AM, such as citrate, isoleucine, and creatine, which may indicate an increased metabolic activity in the mammary gland. Citrate is one of the main components of human milk and it is an intermediate metabolite of the tricarboxylic acid cycle (TCA cycle) and lipid synthesis. These results are consistent with those of Tong et al. (2019), who also reported that the citrate cycle pathway was increased during subclinical mastitis [43] in cows.

The results of the present study also show alteration of the lipid profile due to increased overgrowth of bacteria during mastitis and the immune response that leads to an inflammatory response and nitric oxide-derived oxidative stress in the breast [30,44,45,46].

Levels of total cholesterol and triglycerides are increased in AM milk, whereas levels of esterified cholesterol are significantly increased in both AM and SAM milk. It has been reported that mastitis-associated inflammation may induce dyslipidemia, increasing the levels of blood triglycerides and LDL cholesterol in rats [47]. Since the permeability of the blood–milk barrier is impaired during the process of mastitis [48], levels of cholesterol and triglycerides might increase in mastitis milk due to the transudation of serum-derived products into milk [49].

Concentrations of lysophosphatidylcholine are significantly increased in both AM and SAM milk. Lysophosphatidylcholine is a signaling molecule associated with the inflammatory response, activates macrophages, and enhances phagocytic activity [50]. Thus, increased levels of lysophosphatidylcholine during mastitis are expected and have been previously reported in one study conducted in cows aiming to identify predictive biomarkers of periparturient disease such as mastitis [51].

Phosphatidylcholine is also significantly altered in AM and SAM milk. Phosphatidylcholine is one of the main structural elements of biological membranes and is the most abundant phospholipid component in plasma and in all lipoprotein classes [50]. It has been demonstrated that somatic cell count increases during mastitis due to the migration of immune cells to the breast [31,41,52]. This high concentration of maternal and immune cells may contribute to the increase in phosphatidylcholine. In line with this fact, the levels of plasmalogen are also significantly increased in AM milk. Plasmalogen protects mammalian cells against the reactive oxygen species caused during infections; consequently, human neutrophils, macrophages, and lymphocyte membranes are rich in plasmalogens [53,54] and their increased levels in milk samples during AM may indicate high activity of immune cells in the breast.

Another glycerophospholipid significantly altered during AM in milk samples is phosphatidylethanolamine. Phosphatidylethanolamine is the main component of microbial membranes [55]; consequently, the growth of mastitis-causing bacteria, such as *S. aureus*, may contribute to the increased levels of phosphatidylethanolamine in breast milk samples during mastitis.

PUFAs, including arachidonic acid (AA), eicosapentaenoic acid (EPA) and docosahexaenoic acid (DHA), are important constituents of the cell membrane and precursors of signal transmitters. Metabolites of AA, EPA and DHA act as bioactive molecules known as lipid mediators. Prostaglandins, thromboxane A2 and AA-derived leukotrienes often act as proinflammatory lipid mediators, and their excessive expression leads to the onset or exacerbation of inflammation. On the other hand, AA-derived lipoxins and EPA-derived and DHA-derived resolvins often exhibit anti-inflammatory and pro-resolving actions [56,57]. The increased inflammatory response in the breast in both AM and SAM processes may explain the significantly increased levels of PUFAs in AM and SAM milk samples. In agreement with our results, Nagasaki et al. (2022) reported a significant increase in thromboxane B2 and protectin D1 in milk from individuals with mastitis, and they proposed these compounds as biomarkers of obstructive mastitis [45].

A multi-adjusted logistic regression model was used to evaluate the association of the significant metabolites in the Wilcoxon test with the development of AM and SAM in order to select the most suitable biomarkers of mastitis, both AM and SAM. The results of these analyses reveal that the potential biomarkers to diagnose both AM and SAM are cholesterol, lysophosphatidylcoholine, phosphatidylcholine, plasmalogen, and total polyunsaturated fatty acids. However, only cholesterol showed a strong association for both AM and SAM (OR > 2.5)

The main weakness of our study is that mastitis (both AM and SAM) was only diagnosed clinically and not confirmed with a reliable milk culture because of a lack of consensus on an accurate milk culture method. Hence, some mastitis samples, especially SAM samples, may be misdiagnosed and the mammary inflammation in those samples could be due to other causes of mastitis other than bacterial infection.

## 5. Conclusions

In conclusion, some metabolic alteration in breast milk occurs during a process of both AM and SAM; in particular, acetate, esterified cholesterol, lysophostidylcholine, and PUFAs increased in both AM and SAM process. However, according to the multi-adjusted regression logistic models, the candidate biomarkers for AM and SAM are cholesterol, lysophosphatidylcoholine, phosphatidylcholine plasmalogen, and total polyunsaturated fatty acids. Further studies are needed to evaluate and validate these potential biomarkers of mastitis in an independent and larger population.

## Figures and Tables

**Table 1:** Baseline characteristics of the participants according to study groups.

		CT	AM	SAM	*p*-Value
Age (mean, SD)		34.6 (3.4)	33.3 (2.6)	34.0 (4.2)	0.375
Educational level	No studies	0 (0%)	0 (0%)	0 (0%)	0.605
	Primary studies	0 (0%)	0 (0%)	0 (0%)	
	Secondary studies	1 (5.3%)	0 (0%)	2 (6.3%)	
	Post-secondary non-tertiary education	1 (5.3%)	3 (21.4%)	5 (15.6%)	
	University studies	17 (89.5%)	11 (78.6%)	25 (78.1%)	
Nationality	Spain	19 (100%)	14 (100%)	27 (84.4%)	0.471
	European countries (other than Spain)	0 (0%)	0 (0%)	1 (3.1%)	
	non-EU European countries	0 (0%)	0 (0%)	1 (3.1%)	
	Central and South America	0 (0%)	0 (0%)	0 (0%)	
	North America	0 (0%)	0 (0%)	0 (0%)	
	Africa	0 (0%)	0 (0%)	3 (9.4%)	
	Asia	0 (0%)	0 (0%)	0 (0%)	
	Oceania	0 (0%)	0 (0%)	0 (0%)	
Previous mastitis	No, it is my first time breastfeeding	14 (73.7%)	10 (71.4%)	25 (78.1%)	0.425
	No	0 (0%)	2 (14.3%)	3 (9.4%)	
	Yes	5 (26.3%)	2 (14.3%)	4 (12.5%)	
Autoimmune disease	No	16 (84.2%)	13 (92.9%)	29 (90.6%)	0.685
	Yes	3 (15.8%)	1 (7.1%)	3 (9.4%)	
Anemia	No	16 (84.2%)	10 (71.4%)	23 (71.9%)	0.752
	I do not know	2 (10.5%)	3 (21.4%)	8 (25%)	
	Yes	1 (5.3%)	1 (7.1%)	1 (3.1%)	
Diabetes	No	19 (100%)	14 (100%)	32 (100%)	**-**
	Type I diabetes	0 (0%)	0 (0%)	0 (0%)	
	Type II diabetes	0 (0%)	0 (0%)	0 (0%)	
Suffered from gestational diabetes	No	19 (100%)	13 (92.9%)	29 (90.6%)	0.397
	Yes	0 (0%)	1 (7.1%)	3 (9.4%)	
Thyroid pathology	No	15 (78.9%)	13 (92.9%)	30 (93.8%)	0.227
	Yes	4 (21.1%)	1 (7.1%)	2 (6.3%)	
Smoking habits	I have never smoked	12 (63.2%)	9 (64.3%)	16 (50%)	0.588
	Former smoker	7 (36.8%)	5 (35.7%)	14 (43.8%)	
	Smoker	0 (0%)	0 (0%)	2 (6.3%)	
Antibiotics during pregnancy	No	16 (84.2%)	12 (85.7%)	27 (84.4%)	0.618
	I do not remember	1 (5.3%)	0 (0%)	0 (0%)	
	Yes	2 (10.5%)	2 (14.3%)	5 (15.6%)	
Antibiotics during labor	No	12 (63.2%)	10 (71.4%)	23 (71.9%)	0.618
	I do not know	2 (10.5%)	1 (7.1%)	2 (6.3%)	
	Yes	5 (26.3%)	3 (21.4%)	7 (21.9%)	
Probiotics during pregnancy	No	16 (84.2%)	10 (71.4%)	31 (96.9%)	**0.046**
	Yes	3 (15.8%)	4 (28.6%)	1 (3.1%)	
Birth	C-section	7 (36.8%)	2 (14.3%)	2 (6.3%)	**0.018**
	vaginal	12 (63.2%)	12 (85.7%)	30 (93.8%)	
Current breastfeeding practices	Exclusive breastfeeding	18 (94.7%)	12 (85.7%)	29 (90.6%)	0.675
	Mixed feeding	1 (5.3%)	2 (14.3%)	3 (9.4%)	
Pacifier use	No	12 (63.2%)	12 (85.7%)	21 (65.6%)	0.315
	Yes	7 (36.8%)	2 (14.3%)	11 (34.4%)	
Breast pump use	No	8 (42.1%)	8 (57.1%)	16 (50%)	0.689
	Yes	11 (57.9%)	6 (42.9%)	16 (50%)	
Antibiotics during breastfeeding	No	15 (78.9%)	7 (50%)	24 (75%)	0.130
	I do not remember	1 (5.3%)	0 (0%)	0 (0%)	
	Yes	3 (15.8%)	7 (50%)	8 (25%)	
Antifungals during breastfeeding	No	18 (94.7%)	14 (100%)	31 (96.9%)	0.687
	Yes	1 (5.3%)	0 (0%)	1 (3.1%)	
Analgesics during breastfeeding	No	6 (31.6%)	3 (21.4%)	14 (43.8%)	0.421
	I do not remember	1 (5.3%)	1 (7.1%)	0 (0%)	
	Yes	12 (63.2%)	10 (71.4%)	18 (56.3%)	
Anti-inflammatory use during breastfeeding	No	6 (31.6%)	4 (28.6%)	11 (34.4%)	0.498
	I do not remember	3 (15.8%)	0 (0%)	2 (6.3%)	
	Yes	10 (52.6%)	10 (71.4%)	19 (59.4%)	
Corticoids during breastfeeding	No	19 (100%)	14 (100%)	28 (87.5%)	0.111
	Yes	0 (0%)	0 (0%)	4 (12.5%)	
Probiotics during breastfeeding	No	10 (52.6%)	6 (42.9%)	7 (21.9%)	0.068
	Yes	9 (47.4%)	8 (57.1%)	25 (78.1%)	
Adherence to Mediterranean Diet score (mean, SD)		7.7 (1.8)	8.7 (1.5)	7.9 (1.6)	0.326
Infant age (months, mean, SD)		3.6 (1.5)	3.3 (1.8)	2.8 (1.4)	0.168
Infant weight at birth in g (mean, SD)		3354 (370)	3498 (670)	3271 (691)	0.941
Infant candidiasis	No	18 (94.7%)	14 (100%)	29 (90.6%)	0.740
	Diaper rush	1 (5.3%)	0 (0%)	2 (6.3%)	
	Oral thrush	0 (0%)	0 (0%)	1 (3.1%)	
Infant hospitalization	No	17 (89.5%)	10 (71.4%)	30 (93.8%)	0.101
	Yes	2 (10.5%)	4 (28.6%)	2 (6.3%)	

*p*-values were obtained using nonparametric Kruskal–Wallis test for multiple groups for continuous variables. For categorical variables, *p*-values were obtained using Chi-square test. Significant variables among the three groups (*p*-value < 0.005) are highlighted in bold.

**Table 2:** Concentration of metabolites found in control, AM, and SAM milk samples.

Metabolite	Control Mean (SD)	Acute Mastitis Mean (SD)	*p*-Value *	Subacute Mastitis Mean (SD)	*p*-Value *
2-Oxoglutarate (µmol/L)	3.8 (1.1)	5.4 (3.6)	0.259	3.8 (1.3)	0.947
Acetate (µmol/L)	1.2 (0.6)	2.7 (1.6)	**0.001**	1.5 (0.6)	**0.011**
Alanine (µmol/L)	19.7 (7.4)	24.6 (10.2)	0.204	19.0 (6.1)	0.738
Alloisoleucine (µmol/L)	0.6 (0.3)	0.4 (0.4)	0.307	0.6 (0.4)	0.962
Free cholesterol (C19L) (a.u.)	4.8 (1.2)	5.7 (1.4)	0.066	4.9 (1.1)	0.945
Cadaverine (µmol/L)	5.3 (1.9)	6.1 (2.3)	0.582	5.0 (2.3)	0.548
Choline (µmol/L)	20.2 (10.0)	29.3 (21.9)	0.204	17.9 (10.9)	0.353
Citrate (µmol/L)	92.6 (25.6)	120.9 (41.7)	**0.027**	100.1 (29.7)	0.394
Creatine (µmol/L)	3.6 (2.4)	5.3 (2.0)	**0.049**	4.1 (1.6)	0.343
Creatine Phosphate (µmol/L)	1.1 (0.8)	1.5 (0.8)	0.169	1.4 (0.6)	0.077
Creatinine (µmol/L)	4.2 (0.9)	3.5 (1.2)	0.381	4.4 (1.3)	0.641
Cholesterol (total) (a.u.)	5.3 (1.3)	7.2 (1.4)	**<0.001**	5.7 (1.2)	0.302
Docosahexaenoic acid (a.u.)	1.9 (1.1)	1.3 (0.6)	0.158	2.0 (1.0)	0.562
Esterified colesterol (a.u.)	0.5 (0.3)	1.5 (0.6)	**<0.001**	0.8 (0.4)	**0.012**
Formate (µmol/L)	2.2 (0.8)	2.5 (1.4)	0.931	2.6 (1.2)	0.270
Glutamate (µmol/L)	118.3 (27.3)	134.2 (54.4)	0.416	127.9 (33.6)	0.353
Glutamine (µmol/L)	67.7 (24.2)	49.9 (36.8)	0.353	49.1 (29.2)	**0.004**
Isoleucine (µmol/L)	0.6 (0.4)	1.9 (2.4)	**0.036**	0.7 (0.6)	0.647
Lactate (µmol/L)	10.1 (5.8)	63.2 (93.4)	**0.001**	10.2 (7.0)	0.883
Lactose (µmol/L)	6220.1 (358.7)	6367.1 (1237.8)	0.204	6061.1 (336.3)	0.052
Leucine (µmol/L)	5.3 (1.6)	9.6 (8.4)	0.091	5.2 (1.7)	0.991
Linoleic acid (a.u.)	223.2 (45.4)	236.8 (36.3)	0.359	230.8 (68.7)	0.947
Lysophosphatidylcholine (a.u.)	0.07 (0.04)	0.3 (0.3)	**<0.001**	0.1 (0.04)	**0.028**
O-Phosphocholine (a.u.)	27.5 (16)	25.6 (15.8)	0.957	40.1(17.5)	**0.003**
Phosphatidylcholine (a.u.)	0.8 (0.2)	1.3 (0.6)	**<0.001**	0.9 (0.2)	0.061
Phosphatidylethanolamine (a.u.)	0.7 (1.0)	2.9 (3.3)	**0.030**	0.5 (0.7)	0.062
Phenylalanine (µmol/L)	0.4 (0.3)	1.1 (0.6)	0.193	0.6 (0.4)	0.179
Plasmalogen (a.u.)	193.4 (33.3)	242.9 (27.3)	**<0.001**	235.8 (58)	0.457
Polyunsaturated fatty acids (a.u.)	1.8 (0.9)	2.4 (0.6)	**<0.001**	1.9 (0.7)	**0.002**
Sphingomyelin (a.u.)	45.0 (16.7)	38.83(12.8)	**0.006**	43.8 (11.9)	0.360
sn-Glycero-3-phosphocholine (a.u.)	0.3 (0.2)	0.2 (0.2)	0.341	0.2 (0.1)	0.886
Succinate (µmol/L)	554.5 (67.8)	0.2 (0.2)	0.275	515.6 (57.2)	0.878
Triglycerides (a.u.)	7.2 (3.3)	475.6 (38.7)	**0.001**	5.3 (2.6)	**0.038**
Threonine (µmol/L)	52.8 (54.4)	4 (2.54)	**0.011**	43.4 (59.7)	0.058
Tyrosine (µmol/L)	19.8 (21)	35.5 (51.1)	0.927	22.3 (14.3)	0.679
Valine (µmol/L)	1.2 (0.6)	0.3 (0.2)	0.056	1.5 (0.6)	0.826

* Compared to control samples. Unpaired U Mann–Whitney–Wilcoxon nonparametric test was used due to the low number of participants in each group. Significant *p*-values are highlighted in bold.

**Table 3:** Association of significant metabolites with the development of AM and SAM. Results of the unadjusted and multi-adjusted * logistic regression models.

Metabolite	Model	Acute Mastitis		Subacute Mastitis	
		OR (95% CI)	*p*-Value	OR (95% CI)	*p*-Value
Acetate	Unadjusted	4.5 (1.9–10.5)	**0.001**	1.38 (0.9–1.9)	0.070
	Multi-adjusted	3.9 (1.4–10.8)	**0.020**	1.22 (0.7–2.0)	0.429
Citrate	Unadjusted	1.3 (1.1–1.7)	**0.011**	1.1 (0.9–1.3)	0.182
	Multi-adjusted	1.4 (0.9–2.1)	0.174	1.0 (0.8–1.3)	0.791
Creatine	Unadjusted	6.6 (1.3–32)	**0.026**	2.0 (0.6–6.4)	0.217
	Multi-adjusted	2.2 (0.8–5.7)	0.648	1.3 (0.2–6.6)	0.738
Cholesterol (total)	Unadjusted	6.3 (2.3–17.3)	**0.001**	1.5 (0.7–3.2)	0.298
	Multi-adjusted	14 (3.2–62)	**0.004**	2.6 (1.1–6.6)	**0.045**
Esterified cholesterol	Unadjusted	2.5 (1.8–3.5)	**<0.001**	1.27 (1.02–1.5)	**0.035**
	Multi-adjusted	3.3 (1.9–5.8)	**0.001**	1.3 (0.9–1.7)	0.068
Glutamine	Unadjusted	0.19 (0.02–1.7)	0.153	0.18 (0.03–0.9)	**0.046**
	Multi-adjusted	0.03 (0.003–0.6)	**0.040**	0.4 (0.04–4.2)	0.473
Isoleucine	Unadjusted	3–5 (1.1–11.1)	**0.038**	1 (0.7–1.4)	0.970
	Multi-adjusted	3.6 (0.3–3.6)	0.293	0.8 (0.5–1.4)	0.585
Lactate	Unadjusted	1.7 (1.1–2.6)	**0.024**	1 (0.9–1.04)	0.659
	Multi-adjusted	2.1 (0.9–4.9)	0.108	1.0 (0.9–1.0)	0.841
Lysophosphatidylcholine	Unadjusted	1.2 (1.1–1.5)	**0.003**	1.02 (1.0–1.05)	**0.023**
	Multi-adjusted	1.4 (1.1–1.8)	**0.021**	1.0 (1.0–1.1)	**0.011**
Phosphatidylcholine	Unadjusted	1.7 (1.2–2.3)	**0.002**	1.1 (1.0–1.3)	**0.028**
	Multi-adjusted	2.2 (1.5–3.3)	**0.001**	1.2 (1.1–1.4)	**0.001**
Plasmalogen	Unadjusted	2.0 (1.4–2.7)	**<0.001**	1.2 (1.0–1.5)	**0.031**
	Multi-adjusted	2.9 (1.7–4.8)	**0.002**	1.3 (1.1–1.8)	**0.047**
Polyunsaturated fatty acids	Unadjusted	1.7 (1.4–2.1)	**<0.001**	1.6 (1.2–2.2)	**0.002**
	Multi-adjusted	2.0 (1.7–2.3)	**<0.001**	1.0 (1.0–1.9)	**0.026**
Triglycerides	Unadjusted	0.4 (0.2–0.6)	**<0.001**	0.6 (0.4–0.9)	**0.019**
	Multi-adjusted	0.3 (0.2–0.4)	**<0.001**	0.7 (0.4–1.1)	0.096
O-phosphocoline	Unadjusted	1.0 (0.3–2.8)	0.972	1.1 (1.0–1.3)	**0.004**
	Multi-adjusted	2.3 (0.5–9.5)	0.288	1.1 (1.0–1.3)	0.056
Sphingomyelin	Unadjusted	1.6 (0.8–3.0)	0.127	1.0 (0.6–1.7)	0.762
	Multi-adjusted	2.6 (1.2–5.8)	**0.036**	1.3 (0.7–2.5)	0.328
Threonine	Unadjusted	0.04 (0.005–0.3)	**0.006**	0.1 (0.02–0.9)	**0.041**
	Multi-adjusted	0.05 (0.03–1.4)	0.108	0.3 (0.02–3.4)	0.345

* Logistic regression models were adjusted based on infant ankyloglossia (presence or absence), autoimmune disease, anemia, gestational diabetes, thyroid disease, smoking habit, antibiotics taken during pregnancy, antibiotics administered during birth, antibiotics taken during breastfeeding, antifungals taken during breastfeeding, and probiotics taken during breastfeeding. Significant *p*-values are highlighted in bold.

## Data Availability

The datasets presented in this article are not readily available due to privacy.

## Abbreviations

AM: acute mastitis, SAM: subacute mastitis, CT: control, PLS-DA: Partial Least Squares Discriminant Analysis.
